# Characterizing and Quantifying Arbovirus Transmission by *Aedes aegypti* Using Forced Salivation and Analysis of Bloodmeals

**DOI:** 10.3390/insects12040304

**Published:** 2021-03-30

**Authors:** Megan R. Miller, Madeleine R. Sorensen, Erin D. Markle, Taylor C. Clarkson, Ashley L. Knight, Michelle J. Savran, Brian D. Foy

**Affiliations:** Center for Vector-Borne Infectious Diseases, Department of Microbiology, Immunology and Pathology, Colorado State University, Fort Collins, CO 80523-1685, USA; madeleine.sorensen@colostate.edu (M.R.S.); erin.markle@ucdconnect.ie (E.D.M.); taylorcclarkson@hotmail.com (T.C.C.); knighta@rams.colostate.edu (A.L.K.); Michelle.Savran@colostate.edu (M.J.S.); brian.foy@colostate.edu (B.D.F.)

**Keywords:** arboviruses, transmission, *Aedes aegypti*, saliva

## Abstract

**Simple Summary:**

Mosquitoes transmit hundreds of arboviruses. The ability to estimate the titers of viruses transmitted from infectious mosquitoes to a host is critical. In this study, we evaluated forced salivation techniques to estimate and compare titers of Zika virus and chikungunya virus transmitted by the mosquitoes. We demonstrated that performing forced salivation on mosquitoes after blood feeding might be an efficient way to estimate the virus transmitted during blood feeding. Additionally, by comparing titers of bloodmeals and saliva post-feeding, we showed that mosquitoes re-ingest much of their saliva during artificial blood feeding. The results from this study add new information to understanding and quantifying the transmission of arboviruses.

**Abstract:**

Arbovirus transmission studies are dependent on the ability to estimate the titer of virus transmitted from infectious mosquitoes to a host. There are several methods for estimating virus titer in mosquito saliva, including (1) using forced salivation (FS) whereby the infectious mosquito’s proboscis is forced into a capillary tube containing media to collect and test their saliva for virus, and (2) by quantifying virus expectorated into host tissues or into the blood contained in an artificial feeder immediately after blood feeding. We studied FS and bloodmeals to estimate and compare titers of Zika virus and chikungunya virus transmitted by the mosquito vector *Aedes aegypti*. Infectious virus and viral genomes of both viruses were detected more often from individual mosquitoes using immersion oil for the FS media compared to fetal bovine serum (FBS) plus glycerol, but the FS media had no influence on virus quantification from positive samples. FS virus titers were equivalent when comparing individuals or groups of mosquitoes that never received a blood meal compared to those that were blood fed immediately prior, showing that blood feeding does not influence FS. This suggested that performing FS on mosquitoes after blood feeding might be an efficient way to estimate virus transmitted during blood feeding. However, detecting virus from the blood remaining in an artificial feeder post-blood feeding was mostly unsuccessful relative to quantifying virus from FS of the post-blood fed mosquitoes. In contrast, immunocompromised mice always became infected after being fed on by Zika-infected mosquitoes, even when no infectious virus was detected in their saliva by FS post-blood feed. Due to this discrepancy, we tested the ingested bloodmeals of individual mosquitoes that fed on artificial blood feeders for virus, and compared these to virus in their saliva harvested from FS and to virus in their bodies. These experiments revealed ~50–100 times higher virus titers in the dissected bloodmeals compared to those detected in the same mosquitoes’ saliva, demonstrating how mosquitoes re-ingest much of their saliva during artificial blood feeding, and highlighting a large increase in virus transmission during *Aedes aegypti* blood feeding. Both FS and the dissected bloodmeals of artificially blood-fed mosquitoes showed that the quantity of viral RNA expectorated by mosquitoes was 2–5 logs more than the quantity of infectious virus. The results from this study add critical information to understanding and quantifying the transmission of *Aedes aegypti* arboviruses.

## 1. Introduction

Estimating the titer of arboviruses transmitted by mosquitoes during blood feeding on a host is critical to understand arbovirus transmission, especially by accurately simulating these natural infections in laboratory studies. There are several documented methods for collecting saliva and/or determining the efficiency and titer of virus transmitted from mosquito saliva to a host: (1) forced salivation (FS) of infectious mosquitoes into media contained in a capillary tube and testing the captured saliva for virus, (2) detecting virus in host tissues immediately after infectious mosquitoes blood feed or by later examining host infection or seroconversion rates, and (3) detecting virus in the remaining blood from artificial feeders fed upon by infectious mosquitoes [1,2,3,4,5,6]. It is also possible to detect arboviral nucleic acids transmitted into sugar solutions when wild or laboratory mosquitoes sugar feed on special collection devices. However, this does not estimate virus transmitted to a host and tends to only be a qualitative measure of transmission potential because the number of sugar-feeding mosquitoes and the frequency at which they sugar-feed is often unknown [7]. The three former quantitative methods each have challenges and their success is dependent on mosquito species and virus. Therefore, it is essential to evaluate the effectiveness of these methods on different mosquitoes and virus combinations to determine the best laboratory practices for predicting viral transmission.

Forced salivation is often used as it can determine virus expectorated from a single mosquito and is not dependent on the mosquito blood feeding. Within this method, different media have been used within the capillary tube, the most common being either microscope immersion oil [2,4] or fetal bovine serum (FBS) [8]. FBS was thought to be a better medium to use because it may aid in viral stabilization and preservation, however, placing the mosquito proboscis into FBS is more difficult due to the hydrophobic properties of the mosquito cuticle [4,9]. In this sense, immersion oil is an easier choice to work with. Results with *Aedes albopictus* infected with Venezuelan equine encephalitis virus (VEEV) showed that there was no difference between virus titers when saliva was collected in either immersion oil or FBS using the FS technique [4].

In addition to forced salivation, vertebrate hosts or artificial feeders can be used to estimate the amount of virus being transmitted from an infectious mosquito bite. However, mosquito feeding behaviors are inconsistent, especially in high-containment (BSL-3) laboratory settings because of rapid air exchange and personal protective equipment that limit body heat and odor cues, so there is no way to ensure any one mosquito, or recalcitrant species or strains, will take a blood meal [10]. A study with Eastern equine encephalitis virus (EEEV)-infected *Aedes aegypti* used mouse intracerebral 50% lethal doses to show that the amount of virus transmitted varied from being undetectable to 1.0 × 10^5^ [11]. An additional study with EEEV showed mosquitoes transmitted ~1.0 × 10^3^ PFU as measured by FS collections with immersion oil [12]. Comparing these two results suggested that the quantities of EEEV transmitted during blood feeding and FS collection were approximately equal. However, other studies with different virus-vector pairings have given disparate results. For example, a study that quantified the amount of West Nile virus (WNV) transmitted by *Culex tarsalis* after blood feeding determined that virus transmitted was approximately 600-fold higher than virus transmitted during the FS technique [13], but another study showed similar virus titers transmitted from *Culex pipiens quinquefasciatus* by blood feeding and forced salivation [14]. Taken together, these results show that there is variation in the amount of virus transmitted in saliva that may be dependent on the virus-vector pairing, and that detection methods vary widely in their accuracy and precision.

**Figure 1:**
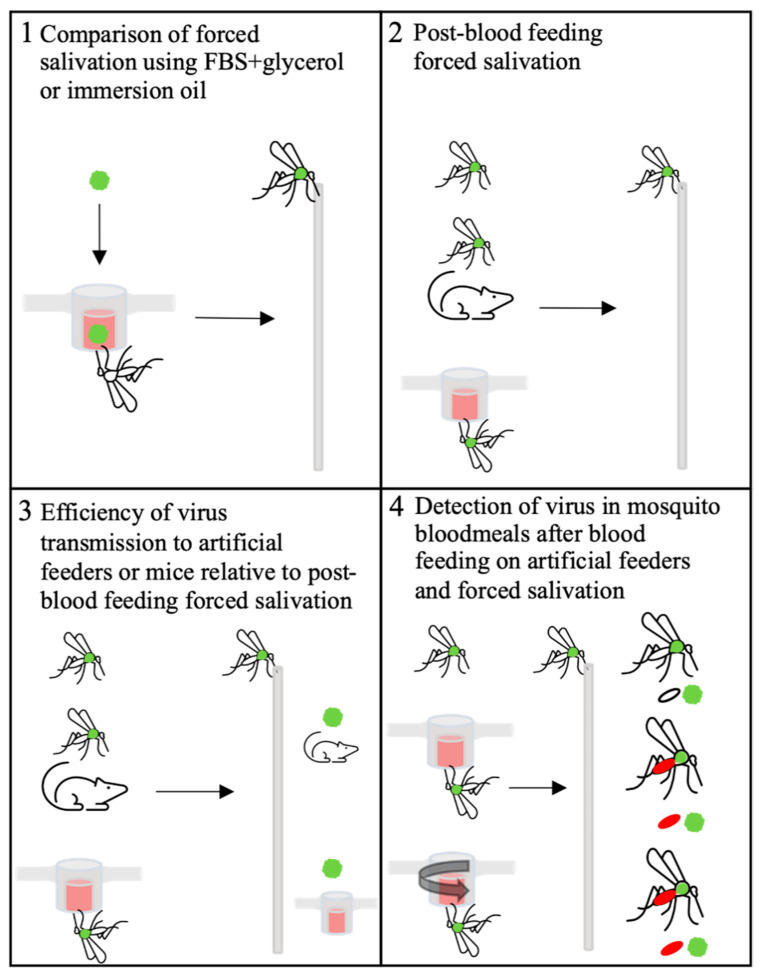
Graphical picture of experiments completed in this study. Green ball represents Zika virus (ZIKV) or chikungunya virus (CHIKV). Green thorax mosquitoes = mosquitoes infected by infectious bloodmeal as shown in panel 1.

Here, we have attempted to quantify virus titers transmitted from *Aedes aegypti* mosquitoes infected with Zika virus (ZIKV) and chikungunya virus (CHIKV). Our efforts examined variations on the FS technique and compared it to virus transmission during blood feeding on animals and artificial feeders, and also to re-ingested virus recovered from bloodmeals dissected out of the mosquitoes (Figure 1). The results from this study add critical information to understanding the transmission of *Aedes aegypti*-borne arboviruses, which are responsible for frequent human disease epidemics across the tropical and sub-tropical areas of the world.

## 2. Materials and Methods

### 2.1. Virus and Cells

African Green Monkey kidney cells (Vero; ATCC #CCL-81) were maintained in Dulbecco’s modified Eagle medium (DMEM) supplemented with 10% fetal bovine serum (DMEM; Gibco Thermo Fisher, FBS; Hyclone, Logan, UT, USA), 2 mM L-glutamine (Gibco Thermo Fisher), 1.5 g/L sodium bicarbonate (Gibco Thermo Fisher), 100 U/mL penicillin (Gibco Thermo Fisher) and incubated at 37 °C in 5% CO_2_. ZIKV strain PRVABC59 (ZIKV-PR; GenBank:KU501215), originally isolated from a human traveler to Puerto Rico in 2015 with three rounds of amplification on Vero cells, was obtained from Dr. Aaron Brault (CDC, Ft. Collins, CO, USA). CHIIKV strain LR2006_OPY1 (GenBank: KT449801.1) was obtained from the University Texas Medical Branch and isolated from outbreak in Reunion Island in 2006 with three rounds of amplification on Vero cells.

### 2.2. Mosquito Infections

*Aedes aegypti* Poza Rica strain mosquitoes were fed an infectious artificial blood meal containing either CHIKV or ZIKV and held for 10–14 days before all subsequent experiments to ensure dissemination of virus to the salivary glands. Infectious bloodmeals were prepared with 1 mL fresh virus contained in the cell-culture supernatant of infected Vero cells and 1 mL of defibrinated calf blood. Back-titering of the bloodmeals ranged between 1 × 10^6^–5 × 10^6^ PFU/mL. Mosquitoes were sorted post blood feeding and were placed in cartons (Huhtamaki, paper food container 64oz) with an organdy cover and given water and a sugar source.

### 2.3. Mice Infection

A129 mice (interferon alpha/beta receptor -/-) 8–12 weeks old were obtained from breeding colony maintained at Colorado State University. Use of mice was approved by the Colorado State University Institutional Animal Care and Use Committee (protocol 15-6677 AA). All procedures were done in accordance with the Guide for the Care and Use of Laboratory Animals of the National Institutes of Health. To infect mice by mosquito bite, *Aedes aegypti* Poza Rica strain mosquitoes were fed an infectious blood meal and held for 14–17 days. Mosquitoes were sorted post blood feeding and 10–20 blood-fed mosquitoes were place in cartons with an organdy cover and given water and a sugar source. To allow the mosquitos to feed on the mice, each mouse was anesthetized using 100 mg/kg ketamine/10 mg/kg xylazine (ketamine: Zetamine from VetOne, xylazine: XylaMed from VET ONE) and placed on the organdy cover of one carton for ~20 min.

### 2.4. Mosquito Sample Collections

Mosquitoes were immediately cold-anesthetized post-blood feeding and their saliva was collected by the FS method described previously [1], briefly their legs and wings were removed and their proboscis was placed into a capillary tube containing either mineral oil or FBS + glycerol at a ratio of 1:1. After 20–30 min, mosquitoes were pulled off the capillary tube and the capillary tube contents were centrifuged into 150 µL of 2x DMEM and held at −80 °C. The bodies were place in a separate tube held at −80 °C to be homogenized in media for later testing. Infections of mosquito bodies and saliva were determined by plaque assay and qRT-PCR. Samples were titrated by Vero cell plaque assay, with a tragacanth gum overlay and staining at day 5 post-cell culture inoculation for ZIKV and day 2 post-cell culture inoculation for CHIKV.

### 2.5. Bloodmeal Dissections

Bloodmeal dissections were done immediately after individual mosquitoes underwent FS. Mosquitoes were dissected on the sides of glass wells partially filled with 200 µL of DMEM supplemented with 10% fetal bovine serum, 2 mM L-glutamine, 1.5 g/L sodium bicarbonate, 100 U/mL penicillin. The midguts were dissected out and spilt open so that the blood meal contents could spill out into the media, and the torn midgut swished into media to extract the whole blood meal. Mock blood meal dissections were performed on non-blood fed mosquitoes exactly the same way but there was no blood meal that could spill out into the media. The media (plus blood meal) was then collected and placed into a tube for later testing, and the body plus torn midgut were placed into another tube and frozen at −80 °C to be homogenized in media for later testing.

### 2.6. RNA Extractions and qRT-PCR

Tubes containing mosquito bodies were homogenized and both saliva and bodies where centrifuged for 5 min at 14,000× *g*. Bloodmeals were collected in 150 µL of DMEM supplemented with 10% fetal bovine serum, 2 mM L-glutamine, 1.5 g/L sodium bicarbonate, 100 U/mL penicillin. RNA was extracted from all samples using the Mag-Bind Viral DNA/RNA 96 kit (Omega Bio-Tek) on the KingFisher Flex Magnetic Particle Processor (Thermo Fisher Scientific). RNA was eluted in 30 µL nuclease-free water. Progmeg GoTaq Probe 1-Step RT-qPCR System Kits were used on RNA extracted from saliva and bodies to quantify CHIKV and ZIKV RNA according to manufacturers’ instructions. Standard cycling condition were followed, one cycle at 45 °C for 15 min, one cycle at 95 °C for 2 min and 40 cycles of 95 °C for 15 s and 60 °C for 1 min. Primers used for CHIKV were Forward (5′-CTTTGAAGTTTCCTTTCGGTGG-3′) and Reverse (5′-ACFFAAFFRAAACTGGTATGG-3′) and Probe-FAM (5′-TCTGCAGCGTCTTTATCCACGGG-3′). Primers used were ZIKV 1086 (5′-CCGCTGCCCAACACAAG-3′) and ZIKV 1162c (5′-CCACTAACGTTCTTTTGCAGACAT-3′). The probe used was ZIKV 1107-FAM (5′-AGCCTACCTTGACAAGCAGTCAGACACTCAA-3′) [15]. Approximately 100 ng of RNA was added to each reaction. Standards were generated for each virus using a full-length viral RNA. RNA was quantified on a Qubit Fluorometer (ThermoFisher Scientific) and diluted to achieve serial 10-fold genome equivalent (GE) dilutions. The standard curve detection of 104–107 GE/reaction had a primer efficiency of 88.62% to 102% with an R^2^ value of 0.971 to 0.997, a slope of −3629 to −3269, and y-intercept = 37.966 to 47.270.

### 2.7. Statistical Analyses

Results in figures were expressed as mean values (horizontal bars) with individual values showing the variance. The statistical details are noted in the figures and/or in the corresponding figure legends. Statistical significance was primarily determined using either Fisher’s exact test, unpaired Student’s *t*-test or a one-way analysis of variance (ANOVA) with a Tukey’s multiple-comparison in GraphPad Prism. Correlation was determined by Spearman’s rank-order correlation in the GraphPad Prism (GraphPad Software, http://www.graphpad.com/faq/viewfaq.cfm?faq=1362 (accessed on 29 March 2021), La Jolla, CA, USA).

## 3. Results

### 3.1. Comparison of Virus Detection from Saliva Collected in FBS + Glycerol or Immersion Oil

**Figure 2:**
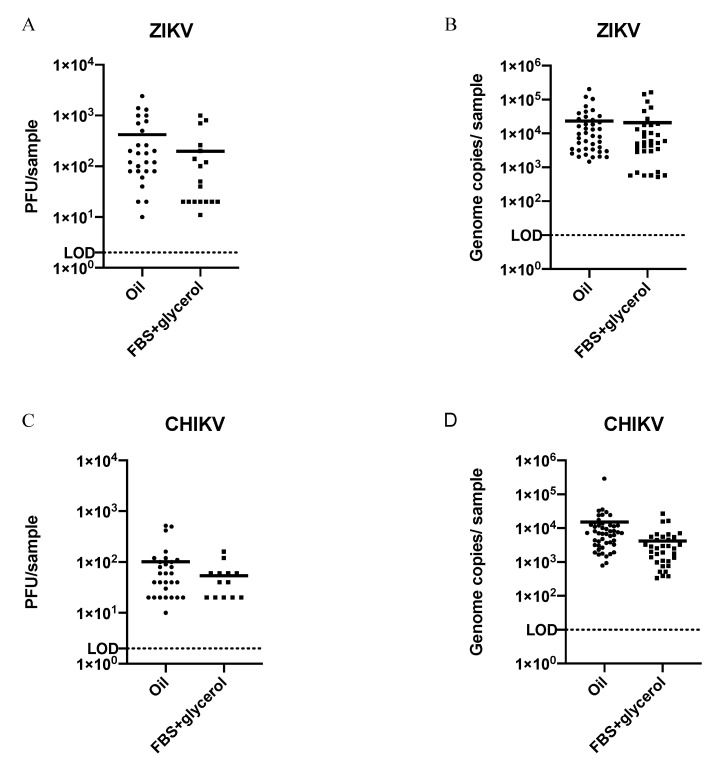
Quantification of virus and viral RNA from virus-positive forced salivations using oil or FBS-glycerol as the collection media; titer and genome copies of virus-positive saliva samples from individual mosquitoes infected with ZIKV (**A**,**B**) or CHIKV (**C**,**D**). LOD = limit of detection. Titer LOD = 2 PFU; genome copies LOD = 10. The means of virus titers or genome copies from saliva collected by either method (horizontal bars), regardless of virus, were not significantly different (un-paired *t*-test; *p* ≥ 0.05).

When ZIKV- and CHIKV-infected *Aedes aegypti* were subjected to FS using two different media, there was a significant difference in the proportion of positive saliva samples from mosquitoes that salivated into oil compared to FBS + glycerol regardless of virus (Table 1). Overall, infectious ZIKV was detected in 31% (30/96) of saliva samples collected in oil, compared to 18% (18/100) of samples collected in FBS + glycerol (*p* ≤ 0.05). The same pattern was seen with detection of infectious CHIKV, with 38% (38/100) of positive saliva samples collected in oil compared to 14% (14/100) from FBS + glycerol (*p* ≤ 0.05). When testing for viral RNA in the same samples, the same pattern was observed, with oil resulting in more positive samples than the FBS + glycerol media for both viruses. However, viral RNA was detected in more samples overall than infectious virus for both viruses. Despite the increased virus prevalence in saliva samples collected using oil, positive samples from both FS media did not significantly differ in the quantity of infectious virus or viral RNA for either virus (Figure 2).

### 3.2. Detection of ZIKV or CHIKV from Mosquito Saliva Post-Blood Feeding

**Figure 3:**
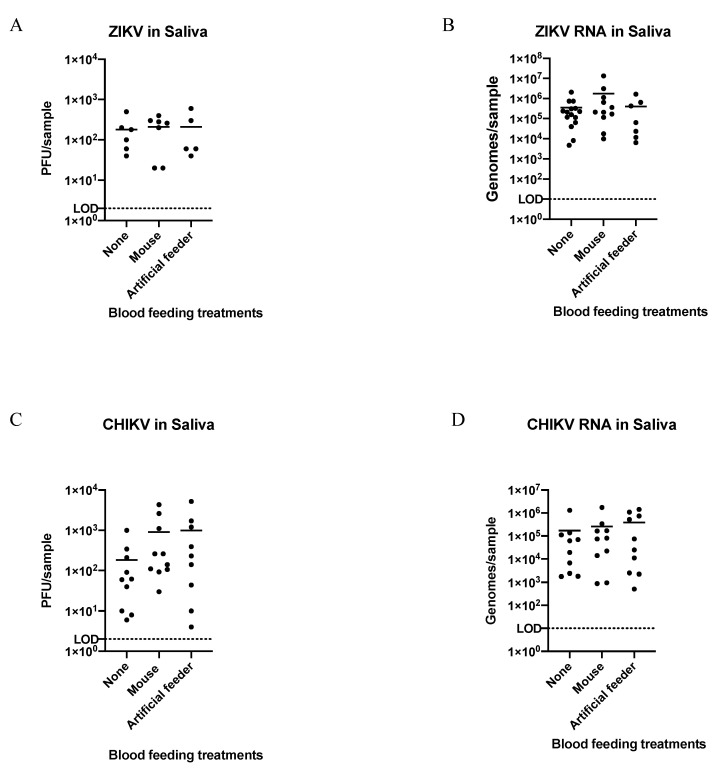
Quantification of ZIKV and CHIKV from groups of virus-positive saliva-samples collected from forced salivation Scheme 2 (**A**,**B**) or CHIKV (**C**,**D**; displayed in Table 3). LOD = limit of detection. Titer LOD = 2 PFU; genome copies LOD = 10. Means of virus titers or genome copies from saliva collected by either method (horizontal bars) were not significantly different (one-way ANOVA and post hoc Tukey test; *p* ≥ 0.05).

Comparisons of virus titers transmitted by ZIKV- and CHIKV-infected *Ae. aegypti* through FS were made post-blood feeding on a mouse or an artificial feeder, in groupings of 1–10 mosquitoes, relative to those that never blood fed (see Figure 1 panel 2 for experimental outline). We strived to group increasing numbers (from 1 to 10) of mosquitoes’ saliva after they blood fed (artificial feeder or mouse) to compare titers from these groups, and made similar groupings from the non-blood feds for equal comparisons (Table 2 and Table 3). There was a small increase in the proportions of groups positive for infectious ZIKV from mosquitoes salivated post-blood feeding (either on an artificial feeder or a mouse) relative to non-blood feds (≥50% vs. 40%, respectively). However, this was not observed when the same samples were tested for viral RNA, and similarly no differences were observed between the proportions of mosquito groups transmitting infectious CHIKV. There were also no significant differences in the quantities of infectious virus or viral RNA expectorated from mosquitoes among the treatment groups (Figure 3). Overall, the quantity of transmitted viral RNA was between 10 and 10,000 times greater than infectious virus in all treatment groups, and there was no correlation between the quantity of virus or viral RNA in the treatment groups and the number of mosquitoes salivated in each group (ZIKV, *p* = 0.692; ZIKV RNA, *p* = 0.121; CHIKV, *p* = 0.576; CHIKV RNA *p* = 0.706, determined by Spearman’s rank-order correlation)

### 3.3. Efficiency of ZIKV or CHIKV Transmission to Artificial Feeders or Mice Relative to Post-Blood Feeding Forced Salivation

Overall, 53 immunocompromised mice were fed upon by groups of ZIKV-infected mosquitoes (between 1–4 individual mosquitoes) and all became infected with ZIKV. Table 4 shows the results from eight of these experiments when FS of the mosquitoes post-blood feeding was unsuccessful in detecting infectious virus. Notably, some mice became infected even after being bit by only one or two mosquitoes from which infectious virus could not be detected and that transmitted low levels of viral RNA (7.2 × 10^2^–6.8 × 10^4^ genome copies) via FS (Table 4). In contrast, infectious virus was never detected, and viral RNA rarely detected, from artificial feeders fed upon by groups of ZIKV- and CHIKV-infected mosquitoes, even though infectious virus and viral RNA was detected in their saliva via FS (Table 5 and Table 6). These data highlighted the sensitivity of immunocompromised mice for detecting transmission of potentially low titers of ZIKV from *Ae. aegypti*, but also highlighted the surprising insensitivity of artificial feeder blood for detecting transmitted virus (both ZIKV and CHIKV) from *Ae. aegypti*. Considering that quantification of viral RNAs from FS of both groups of blood fed mosquitoes was roughly equal, the combined data suggested that some factor was inhibiting our ability to detect arbovirus transmission by *Ae. aegypti* from the blood in the artificial feeders. We hypothesized that this inhibitory factor was mosquito re-ingestion of their saliva, and thus the virus they expectorated, during blood feeding on artificial feeders.

### 3.4. Detection of ZIKV or CHIKV in Mosquito Bloodmeals

**Figure 4:**
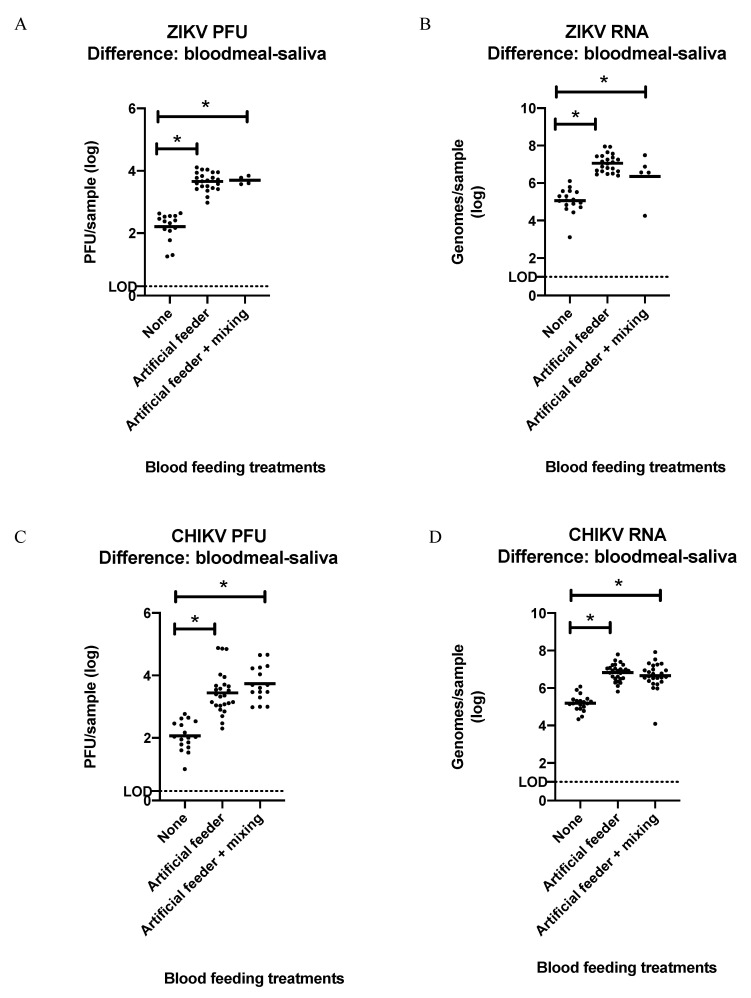
Log transformed difference between ZIKV and CHIKV detected in the bloodmeals and saliva of individual infected mosquitoes after they were given different blood feeding treatments. (**A**) Infectious ZIKV mean difference: None = 2.21, Artificial feeder = 3.65, Artificial feeder + mixing = 3.69. (**B**) ZIKV genome copies mean difference: none = 5.05, artificial feeder = 7.06, artificial feeder + mixing = 6.35. (**C**) Infectious CHIKV mean difference: none = 2.06, artificial feeder = 3.41, artificial feeder + mixing = 3.73. (**D**) CHIKV genome copies mean difference: none = 5.19, artificial feeder = 6.82, artificial feeder + mixing = 6.65. Graphs show both PFUs determined by plaque assay and RNA determined by qRT-PCR. Titer LOD = 2 PFU; genome copy LOD = 10 genomes. Means are horizontal bars (one-way ANOVA and post hoc Tukey test; * *p* ≤ 0.05).

To test our hypothesis of re-ingested virus during blood feeding, we dissected and tested the bloodmeals of ZIKV- and CHIKV-infected mosquitoes after they fed on artificial feeders and then underwent forced salivation. As the process of dissecting out the blood meal results in some contamination of virus into the dissecting media from the hemolymph and midgut tissue of these infected mosquitoes, mock blood meal dissections were performed as treatment controls on groups of mosquitoes that did not get a second bloodmeal. Approximately 100-fold more infectious ZIKV or ZIKV RNA, as well as infectious CHIKV or CHIKV RNA, was detected in the bloodmeals from individual mosquitoes relative to the treatment controls (Supplemental Tables S1 and S2). These results were consistent between groups that fed on artificial feeders that were manipulated so that the blood in them was mixed during the blood feed, compared to those that were not. Importantly, however, a similar increase was not observed in the virus/viral RNA detected in the saliva from these same treatment groups, nor from the bodies of mosquitoes in these same treatment groups (Supplemental Tables S1 and S2). Overall, the log transformed mean differences of virus quantified between the bloodmeal and saliva collected from ZIKV-infected mosquitoes given blood feeding treatments were significantly different from the control treatment (none = 2.214 PFU/5.059 genome copies (gc), artificial feeder = 3.656 PFU/7.064 gc, artificial feeder + mixing = 3.699 PFU/6.350 gc) (Figure 4A,B; one-way ANOVA and post hoc Tukey test; *p* ≤ 0.05). A similar difference was seen with CHIKV-infected mosquitoes (none = 2.067 PFU/5.195 genome copies (gc), artificial feeder = 3.443 PFU/6.832 gc, artificial feeder + mixing = 3.739 PFU/6.659 gc) (Figure 4C,D; one-way ANOVA and post hoc Tukey test; *p* ≤ 0.05).

## 4. Discussion

We used different experimental procedures and methods to estimate the titer of virus transmitted by *Aedes aegypti* mosquitoes after being infected with either ZIKV or CHIKV. Different medias have been previously evaluated for FS, including immersion oil and a mixture of 1:1 FBS + glycerol, as well as blood and other medias [2,4,6,8,16,17,18,19]. Results from these studies showed that the effectiveness of the media depends on mosquito and virus species, but no other study compared these methods with *Ae. aegypti* infected with CHIKV or ZIKV. We found no evidence that FBS + glycerol aided in viral stabilization and preservation as was previously suggested [4,9] and no difference in titers from ZIKV- or CHIKV-positive samples, however, infectious saliva collected using oil resulted in significantly more positive samples from individual mosquitoes. The use of oil with FS is also easier to perform because the mosquitoes are drawn into the capillary tube via the similar hydrophobic properties of the mosquito’s cuticle and the oil, which draws saliva out of the salivary glands into the oil. Additionally, proof of successful saliva capture can be observed, and its quantity estimated, because the hydrophobic oil and aqueous saliva do not mix [20]. In the same paper, Sanchez-Vargas et al. estimated that individual *Aedes aegypti* expectorated a mean of 6.8 nL using oil-based FS and observed no correlation of CHIKV titers with the saliva volume expectorated. Another potential benefit of oil-based FS is that the mosquitoes are unable to re-ingest their own saliva. It has been previously shown that anopheline mosquitoes will re-ingest many of the *Plasmodium* sporozoites they deposit in the host when blood feeding [21]. It follows that saliva re-ingestion could also influence the virus detection success in FS.

FS has been used as the standard method to determine transmission of mosquito-borne arboviruses [1,2,4,5], but we are not aware of any examination of forced salivation on mosquitoes that blood fed immediately prior. Given the large variances in virus transmission by any one mosquito (Table 2 and Table 3 and the supplemental tables), we wanted to know if virus transmission estimates using FS were different if performed immediately after they imbibed a second blood meal relative to estimates using FS from sibling mosquitoes never given a second blood meal. If there were no differences, one would be able to estimate the amount of virus transmitted in laboratory experiments, or even natural experiments in the field, using FS immediately after one or more mosquitoes took a blood meal on a host. For example, one could capture indoor resting blood fed mosquitoes from the walls of a house, perform FS on them immediately post-capture, and reliably estimate the titer that they may have just transmitted to the people in the house whom they bit. We used groups of mosquitoes (1–10 mosquitoes/group) that were given three different blood feeding treatments (none, blood fed on a mouse, blood fed on an artificial feeder) and demonstrated that infectious virus and viral RNA titers determined from FS were not different between the treatments. Furthermore, the ratios of infectious virus to viral RNA quantified were not different between the treatment groups. Each treatment group showed ~100–10,000 times more viral RNA than infectious virus as has been reported in many other studies using FS on unfed mosquitoes alone [2,22].

To compare our post-blood feeding FS data with the quantity of virus transmitted during the blood feed, we analyzed outcomes of ZIKV-infected mosquitoes that blood fed on immunocompromised mice, and virus transmitted to artificial feeders after ZIKV- or CHIKV-infected mosquitoes fed on them. Mice became infected after mosquito feeding even when no infectious virus was detected in the mosquito saliva post feeding. Our limit of detection is 2 PFU and 10 genomes copies; however, we never detected anything under 10 PFU. Based on these results, we can assume these mice can become infected with less than 10 PFU transmitted by mosquitoes blood feeding on them. When examining the blood remaining in the artificial feeders, however, infectious virus was never recovered, and viral RNA was only recovered in four out of nine ZIKV groups and one out of nine CHIKV groups. This observation could be explained in at least two non-exclusive hypotheses. It may be that live virus was quickly inactivated and viral nucleic acid sequences were destroyed by proteases and nucleases in the artificial blood meal, making their detection difficult by plaque assay and qRT-PCR, respectively. However, this seems unlikely given that we rarely record a drop in virus titer of the original blood meal used to first infect the mosquitoes when it is ‘back-titered’ after sitting in the artificial feeder for ~30 min during the blood feed. Another possibility is that virus expelled with the saliva into the artificial blood meal may be immediately re-ingested through the suction force needed to bring blood into the food canal.

To address the latter hypothesis, we dissected out bloodmeals from the infected mosquito midguts after they were given different blood feeding treatments and then were processed with FS. For one of the blood feeding treatments, blood was pipetted up and down in the blood feeder during the time of feeding to determine if blood mixing might counteract the re-ingestion of a mosquito’s own expectorated virus during blood feeding. Compared to the control treatment (mock dissection of bloodmeals from empty midguts), significantly more virus and viral RNA was recovered from the bloodmeals of the two blood feeding treatment groups, and the mixing of the blood in the artificial feeder did not influence this. This indicated that mosquitoes re-ingest much of their expectorated virus while feeding on the artificial feeder and that poor detection of virus in the remaining blood from artificial feeding is likely due to re-ingestion. As each mosquito dissected of its bloodmeal was also processed via FS, we could determine the difference of virus titers between the bloodmeal and saliva to estimate the quantity of virus transmitted during blood feeding. The estimate was consistent between *Ae. aegypti* transmitting either ZIKV or CHIKV and between quantities of infectious virus or viral RNA detected; between 50–100 times more virus is secreted during blood feeding than is detected in FS performed immediately after blood feeding, suggesting a large increase in virus transmission during blood feeding. One limitation of these data are that they are estimates of virus transmission by *Ae. aegypti* derived from blood feeding on artificial feeders, which may not accurately reflect what occurs during blood feeding on live hosts, including transmission during probing but not blood feeding [23]. However, blood feeding on live hosts results in diverse outcomes. Mosquitoes will capillary feed by either fully cannulating capillaries, or just pierce the capillary at a right angle with the tip of the labrum, or sometimes might only nick a capillary and perform ‘pool feeding’ on the blood that pools into the interstitial space of the dermis [24]. Each of these methods are likely to result in differing quantities of saliva/virus deposited as well as being re-ingested back into the blood meal. As such, artificial feeders may be a more consistent blood source for this estimation. In natural blood feeding experiments, Secundino et al. determined that the ZIKV cDNA ranged from 2.0 × 10^2^–2.1 × 10^10^ when the mouse ear tissue was immediately removed and homogenized after being fed on by ZIKV-infected *Ae. aegypti* mosquitoes [25]. In our study, this quantity is more comparable to the RNA loads in bloodmeals rather than saliva, indicating that mosquitoes re-ingest much of their saliva during natural blood feeding.

Studies using different combination of viruses and mosquito species have evaluated the use of vertebrate hosts or artificial feeders to estimate the amount of virus being transmitted from an infectious mosquito and found varying results [11,12,13,14]. Our data allow for estimation of the amount of ZIKV or CHIKV from an infectious *Ae. aegypti* mosquito by performing FS on it immediately post-blooding, quantifying infectious virus or viral RNA and then multiplying the titer determined by ~50–100. Quantifying infectious virus ensures measurement of true infectious units, but it is clearly of low sensitivity and so simultaneously quantifying viral RNA will give the best estimates of transmission dose. More experiments will be necessary to determine if the increase in virus transmission during blood feeding relative to FS we observed is because of (a) more saliva being released by *Ae. aegypti* or (b) more virus being released from the salivary glands, or both. Similarly, results from this study should be replicated with other arbovirus vectors to determine if they are consistent across mosquito species. Overall, the methods developed here can be used as a better way to estimate the titer of arboviruses transmitted by blood feeding *Ae. aegypti* and may be valuable for similar estimations with other mosquitoes.

## Figures and Tables

**Table 1:** Proportions of saliva collections from individual mosquitoes that were ZIKV- or CHIKV-positive using either qRT-PCR or plaque assays when force salivated using oil or FBS-glycerol as the collection media.

Virus	Collection Media	qRT-PCR	*p*-Value	Plaque Assay	*p*-Value
ZIKV	FBS + glycerol	42% (42/100)	* 0.0170	18% (18/100)	* 0.0455
	Oil	65% (62/96)		31% (30/96)	
CHIKV	FBS + glycerol	47% (46/98)	* ≤0.0001	14% (14/98)	* 0.0002
	Oil	82% (82/100)		38% (38/100)	

Data are the sum of two biological replicates. *p*-value from Fisher’s exact text; * indicates *p* ≤ 0.05.

**Table 2:** Detection of ZIKV from forced salivation samples of groups of infected mosquitoes that underwent different blood feeding treatments.

Blood Feeding Treatments	Group ID	Number of Mosquitoes That Blood Fed	Titer (PFU/Sample)	Genome Copies/Sample
None	1	1	0	4.0 × 10^4^
None	2	1	0	2.1 × 10^5^
None	3	2	1.0 × 10^2^	4.7 × 10^3^
None	4	2	0	8.1 × 10^3^
None	5	3	0	1.1 × 10^5^
None	6	3	4.0 × 10^1^	6.3 × 10^5^
None	7	5	2.0 × 10^2^	3.1 × 10^5^
None	8	5	0	2.6 × 10^6^
None	9	7	1.8 × 10^2^	2.3 × 10^5^
None	10	7	0	1.5 × 10^5^
None	11	10	0	2.2 × 10^5^
None	12	10	0	7.3 × 10^5^
None	13	7	5.0 × 10^2^	7.3 × 10^5^
None	14	10	0	1.1 × 10^5^
None	15	10	6.0 × 10^1^	3.2 × 10^5^
Mouse	1	1	2.8 × 10^2^	1.3 × 10^7^
Mouse	2	1	0	9.8 × 10^3^
Mouse	3	2	4.0 × 10^2^	2.0 × 10^5^
Mouse	4	2	0	2.0 × 10^5^
Mouse	5	3	2.6 × 10^2^	6.4 × 10^5^
Mouse	6	3	2.0 × 10^1^	1.7 × 10^4^
Mouse	7	5	3.0 × 10^2^	3.6 × 10^5^
Mouse	8	5	0	1.1 × 10^5^
Mouse	9	7	0	1.7 × 10^5^
Mouse	10	7	2.0 × 10^1^	3.1 × 10^6^
Mouse	11	7	2.0 × 10^2^	1.1 × 10^6^
Artificial feeder	1	1	3.0 × 10^2^	6.4 × 10^4^
Artificial feeder	2	1	6.0 × 10^2^	1.6 × 10^6^
Artificial feeder	3	3	6.0 × 10^1^	6.4 × 10^5^
Artificial feeder	4	2	0	6.3 × 10^3^
Artificial feeder	5	1	0	1.1 × 10^4^
Artificial feeder	7	1	4.0 × 10^1^	2.2 × 10^4^
Artificial feeder	10	3	6.0 × 10^1^	4.2 × 10^5^

**Table 3:** Detection of CHIKV from forced salivation samples of groups of infected mosquitoes that underwent different blood feeding treatments.

Blood Feeding Treatments	Group ID	Number Blood Feed Mosquitoes	Titer (PFU/mL)	Genome Copies
None	1	1	0	0
None	2	1	1.0 × 10^1^	2.4 × 10^3^
None	3	2	6.2 × 10^1^	6.7 × 10^3^
None	4	2	8	1.7 × 10^3^
None	5	3	6	1.8 × 10^3^
None	6	3	3.4 × 10^2^	6.2 × 10^4^
None	7	5	9.0 × 10^1^	1.9 × 10^4^
None	8	5	1.0 × 10^3^	1.3 × 10^6^
None	9	7	3.2 × 10^1^	1.3 × 10^5^
None	10	7	8.8 × 10^1^	6.9 × 10^4^
None	11	4	2.01 × 10^2^	1.1 × 10^5^
Mouse	1	1	0	8.7 ×10^2^
Mouse	2	1	3.0 × 10^1^	9.5 ×10^2^
Mouse	3	2	1.0 × 10^2^	1.4 × 10^4^
Mouse	4	2	1.1 × 10^3^	3.4 × 10^5^
Mouse	5	3	1.4 × 10^2^	0
Mouse	6	3	4.3 × 10^3^	7.5 × 10^5^
Mouse	7	5	2.6 × 10^2^	1.6 × 10^5^
Mouse	8	5	2.6 × 10^2^	1.3 × 10^5^
Mouse	9	7	1.1 × 10^2^	8.0 × 10^4^
Mouse	10	7	2.6 × 10^3^	1.7 × 10^6^
Mouse	11	6	9.2 × 10^1^	2.2 × 10^4^
Artificial feeder	1	1	0	0
Artificial feeder	2	1	4	2.2 × 10^4^
Artificial feeder	3	2	0	5.3 × 10^2^
Artificial feeder	4	2	1.4 × 10^2^	1.1 × 10^4^
Artificial feeder	5	3	1.0 × 10^1^	2.4 × 10^3^
Artificial feeder	6	3	2.3 × 10^2^	7.4 × 10^4^
Artificial feeder	7	5	1.2 × 10^3^	7.4 × 10^5^
Artificial feeder	8	5	5.2 × 10^3^	1.4 × 10^6^
Artificial feeder	9	7	1.7 × 10^3^	1.1 × 10^6^
Artificial feeder	10	7	3.9 × 10^2^	5.2 × 10^5^
Artificial feeder	11	5	4.4 × 10^1^	2.4 × 10^4^

**Table 4:** Detection of ZIKV from forced salivations after infected mosquitoes blood fed on immunocompromised mice.

Mouse ID	Number of Mosquitoes That Blood Fed	Pooled Saliva-Titer (PFU)	Pooled Saliva-Genome Copies	Mouse Became Infected as Determine by Viral RNA Detection in Tissue/Blood?
080	4	0	2.8 × 10^5^	Yes
086	2	0	2.0 × 10^4^	Yes
095	1	0	7.2 × 10^2^	Yes
109	2	0	2.0 × 10^4^	Yes
165	3	0	1.3 × 10^3^	Yes
166	1	0	5.0 × 10^3^	Yes
169	2	0	6.8 × 10^4^	Yes
174	3	0	3.3 × 10^3^	Yes

Pooled saliva titers were determined by plaque assay, limit of detection (LOD) = 2 PFU. Pooled saliva genome copies were determined by qRT-PCR, LOD = 10 genome copies. In total, 53 mice became infected after being bitten by ZIKV-infected mosquitoes. Eight of these mice (shown above) became infected even though the mosquitoes that bit them had undetectable titers of virus in their saliva as measured by FS post-blood feeding.

**Table 5:** Detection of ZIKV from forced salivations and from the blood remaining in the artificial feeders after infected mosquitoes blood fed on them.

Group Number	Number of Mosquitoes that Blood Fed	Pooled Saliva-Titer (PFU)	Pooled Saliva-Genome Copies	Remaining Blood in the Artificial Feeder-Titer (PFU)	Remaining Blood in the Artificial Feeder-Genome Copies
1	2	8.0 × 10^1^	1.1 × 10^5^	0	2.1 × 10^3^
2	6	3.0 × 10^1^	1.6 × 10^4^	0	7.0 × 10^2^
3	4	1.8 × 10^1^	9.5 × 10^3^	0	0
4	5	1.2 × 10^2^	2.2 × 10^5^	0	3.5 × 10^3^
5	9	4.0 × 10^1^	1.9 × 10^5^	0	0
6	2	5.0 × 10^1^	4.5 × 10^5^	0	0
7	7	2.3 × 10^2^	1.9 × 10^5^	0	0
8	5	1.0 × 10^2^	1.2 × 10^6^	0	0
9	3	2.3 × 10^2^	9.0 × 10^4^	0	1.7 × 10^3^

Pooled saliva titers determined by plaque assay, limit of detection (LOD) = 2 PFU. Pooled saliva genome copies determined by qRT-PCR LOD = 10 genome copies.

**Table 6:** Detection of CHIKV from forced salivations and from the blood remaining in the artificial feeders after infected mosquitoes blood fed on them.

Group Number	Number of Mosquitoes That Blood Fed	Pooled Saliva-Titer (PFU)	Pooled Saliva-Genome Copies	Remaining Blood in the Artificial Feeder-Titer (PFU)	Remaining Blood in the Artificial Feeder-Genome Copies
1	2	2.1 × 10^2^	1.1 × 10^5^	0	0
2	11	3.7 × 10^3^	1.8 × 10^6^	0	0
3	10	2.0 × 10^2^	9.5 × 10^4^	0	0
4	7	3.3 × 10^2^	5.0 × 10^4^	0	2.8 × 10^3^
5	6	2.1 × 10^2^	1.0 × 10^5^	0	0

Pooled saliva titers determined by plaque assay, limit of detection (LOD) = 2 PFU. Pooled saliva genome copies determined by qRT-PCR LOD = 10 genome copies.

## Data Availability

The data presented in this study are contained within the article and supplemental data. Supplemental data available in Supplemental Tables S1 and S2.

## Supplementary Materials

The following are available online at https://www.mdpi.com/article/10.3390/insects12040304/s1, Table S1: Quantification of virus from ZIKV-infected mosquitoes given different blood feeding treatments, then force salivated and dissected of their bloodmeals. Table S2: Quantification of virus from CHIKV-infected mosquitoes given different blood feeding treatments, then force salivated and dissected of their bloodmeals.

## References

[1] Heitmann A., Jansen S., Lühken R., Leggewie M., Schmidt-Chanasit J., Tannich E. (2018). Forced Salivation As a Method to Analyze Vector Competence of Mosquitoes. J. Vis. Exp..

[2] Colton L., Biggerstaff B.J., Johnson A., Nasci R.S. (2005). Quantification of West Nile Virus in Vector Mosquito Saliva. J. Am. Mosq. Control Assoc..

[3] Anderson S.L., Richards S.L., Smartt C.T. (2010). A simple method for determining arbovirus transmission in mosquitoes. J. Am. Mosq. Control Assoc..

[4] Smith D.R., Carrara A.-S., Aguilar P.V., Weaver S.C. (2005). Evaluation of methods to assess transmission potential of venezuelan equine encephalitis virus by mosquitoes and estimation of mosquito saliva titers. Am. J. Trop. Med. Hyg..

[5] Gubler D.J., Rosen L. (1976). A Simple Technique for Demonstrating Transmission of Dengue Virus by Mosquitoes without the Use of Vertebrate Hosts. Am. J. Trop. Med. Hyg..

[6] Hurlbut H.S. (1966). Mosquito Salivation and Virus Transmission. Am. J. Trop. Med. Hyg..

[7] Danforth M.E., Reisen W.K., Barker C.M. (2018). Detection of Arbovirus Transmission via Sugar Feeding in a Laboratory Setting. J. Med. Entomol..

[8] Mores C.N., Turell M.J., Dohm D.J., Blow J.A., Carranza M.T., Quintana M. (2007). Experimental Transmission of West Nile Virus by *Culex nigripalpus* from Honduras. Vector-Borne Zoonotic Dis..

[9] Grosz D.D., van Geelen A., Gallup J.M., Hostetter S.J., Derscheid R.J., Ackermann M.R. (2014). Sucrose stabilization of Respiratory Syncytial Virus (RSV) during nebulization and experimental infection. BMC Res. Notes.

[10] Ribeiro J.M.C. (2000). Blood-feeding in mosquitoes: Probing time and salivary gland anti-haemostatic activities in representatives of three genera (Aedes, Anopheles, Culex). Med. Vet.Entomol..

[11] Ohambeblain E.W., Kis8ling B.E., Sike8 B.K. (1954). Studies on the North American Arthropod-Borne Encephalitides vh. Estimation of Amount of Eastern Equine Encephalitis Vxbus Inoculated by Infected Aede8 Aeoypti. Am. J. Epidemiol..

[12] Weaver S.C., Scott T.W., Lorenz L.H. (1990). Patterns of Eastern Equine Encephalomyelitis Virus Infection in Culiseta melanura (Diptera: Culicidae). J. Med. Entomol..

[13] Styer L.M., Kent K.A., Albright R.G., Bennett C.J., Kramer L.D., Bernard K.A. (2007). Mosquitoes Inoculate High Doses of West Nile Virus as They Probe and Feed on Live Hosts. PLoS Pathog..

[14] VanLandingham D.L., Beasley D., Klingler K., Higgs S., Huang J., Fair J., Schneider B.S., Hamilton P. (2004). Real-time reverse transcriptase–polymerase chain reaction quantification of west nile virus transmitted by culex pipiens quinquefasciatus. Am. J. Trop. Med. Hyg..

[15] Lanciotti R.S., Kosoy O.L., Laven J.J., Velez J.O., Lambert A.J., Johnson A.J., Stanfield S.M., Duffy M.R. (2008). Genetic and serologic properties of Zika virus associated with an epidemic, Yap State, Micronesia, 2007. Emerg. Infect. Dis..

[16] Goddard L.B., Roth A.E., Reisen W.K., Scott T.W. (2002). Vector Competence of California Mosquitoes for West Nile virus. Emerg. Infect. Dis..

[17] Cornel A.J., Jupp P.G. (1989). Comparison of three methods for determining transmission rates in vector competence studies with Culex univittatus and West Nile and Sindbis viruses. J. Am. Mosq. Control Assoc..

[18] Aitken T.H.G. (1977). An In vitro feeding technique for artificially demonstrating virus transmission by mosquitoes. Mosq. News.

[19] Beaty B.J., Aitken T.H.G. (1979). In vitro transmission of yellow fever virus by geographic strains of Aedes aegypti. Mosq. News.

[20] Sanchez-Vargas I., Harrington L.C., Black W.C., Olson K.E. (2019). Analysis of Salivary Glands and Saliva from Aedes albopictus and Aedes aegypti Infected with Chikungunya Viruses. Insects.

[21] Kebaier C., Vanderberg J.P. (2006). Re-ingestion of Plasmodium berghei sporozoites after delivery into the host by mosquitoes. Am. J. Trop. Med. Hyg..

[22] Robison A., Young M.C., Byas A.D., Rückert C., Ebel G.D. (2020). Comparison of Chikungunya Virus and Zika Virus Replication and Transmission Dynamics in Aedes aegypti Mosquitoes. Am. J. Trop. Med. Hyg..

[23] Yamamoto D.S., Yokomine T., Sumitani M., Yagi K., Matsuoka H., Yoshida S. (2013). Visualization and live imaging analysis of a mosquito saliva protein in host animal skin using a transgenic mosquito with a secreted luciferase reporter system. Insect Mol. Biol..

[24] Choumet V., Attout T., Chartier L., Khun H., Sautereau J., Robbe-Vincent A., Brey P.T., Huerre M., Bain O. (2012). Visualizing Non Infectious and Infectious Anopheles gambiae Blood Feedings in Naive and Saliva-Immunized Mice. PLoS ONE.

[25] Secundino N.F.C., Chaves B.A., Orfano A.S., Silveira K.R.D., Rodrigues N.B., Campolina T.B., Nacif-Pimenta R., Villegas L.E.M., Silva B., Lacerda M.V.G. (2017). Zika virus transmission to mouse ear by mosquito bite: A laboratory model that replicates the natural transmission process. Parasites Vectors.

