# Supplementary material for: Characterizing and Quantifying Arbovirus Transmission by Aedes aegypti Using Forced Salivation and Analysis of Bloodmeals
Source: Insects. 2021 Mar 30;12(4):304. doi: 10.3390/insects12040304 (PMC8065531; doi:10.3390/insects12040304)
Supplement: Supplementary file 1 [file insects-12-00304-s001.zip › Insects_Supplemental_Tables.docx]

| **Supplemental Table 1.** Quantification of virus from ZIKV-infected mosquitoes given different blood feeding treatments, then force salivated and dissected of their bloodmeals. | | | | | | | | | |
| --- | --- | --- | --- | --- | --- | --- | --- | --- | --- |
| **Treatment** | **Mosquito**  **number** | **Bodies** | | **Saliva** | | **Bloodmeal** | | **Difference:**  **bloodmeal-saliva** | |
| None |  | Titer | Genome Copy | Titer | Genome Copy | Titer | Genome Copy | Titer | Genome Copy |
|  | 1 | 1.60E+05 | 8.20E+08 | 4.00E+00 | 6.70E+03 | 1.40E+02 | 2.10E+05 | 1.36E+02 | 2.03E+05 |
|  | 2 | 1.30E+05 | 3.50E+08 | 2.00E+00 | 9.90E+03 | 1.50E+02 | 1.30E+06 | 1.48E+02 | 1.29E+06 |
|  | 3 | 4.00E+04 | 6.00E+08 | 0.00E+00 | 9.30E+03 | 4.30E+02 | 3.80E+05 | 4.30E+02 | 3.71E+05 |
|  | 4 | 5.00E+04 | 2.70E+08 | 0.00E+00 | 0.00E+00 | 2.90E+02 | 3.30E+05 | 2.90E+02 | 3.30E+05 |
|  | 5 | 5.00E+04 | 8.00E+08 | 2.00E+00 | 6.20E+03 | 2.00E+01 | 4.80E+04 | 1.80E+01 | 4.18E+04 |
|  | 6 | 1.30E+05 | 4.30E+08 | 0.00E+00 | 1.10E+04 | 2.00E+01 | 8.00E+04 | 2.00E+01 | 6.90E+04 |
|  | 7 | 1.30E+05 | 2.00E+08 | 0.00E+00 | 5.80E+03 | 2.40E+02 | 3.30E+04 | 2.40E+02 | 2.72E+04 |
|  | 8 | 6.00E+02 | 1.20E+08 | 0.00E+00 | 1.40E+04 | 1.20E+02 | 6.30E+05 | 1.20E+02 | 6.16E+05 |
|  | 9 | 0.00E+00 | 1.50E+03 | 0.00E+00 | 0.00E+00 | 0.00E+00 | 0.00E+00 | 0.00E+00 | 0.00E+00 |
|  | 10 | 0.00E+00 | 4.50E+08 | 2.00E+00 | 0.00E+00 | 4.40E+02 | 3.80E+05 | 4.38E+02 | 3.80E+05 |
|  | 11 | 6.00E+04 | 6.20E+04 | 0.00E+00 | 0.00E+00 | 0.00E+00 | 1.30E+03 | 0.00E+00 | 1.30E+03 |
|  | 12 | 1.90E+05 | 3.10E+08 | 1.20E+01 | 1.70E+04 | 3.70E+02 | 1.30E+05 | 3.58E+02 | 1.13E+05 |
|  | 13 | 1.50E+05 | 5.70E+08 | 0.00E+00 | 2.50E+04 | 6.00E+01 | 7.70E+04 | 6.00E+01 | 5.20E+04 |
|  | 14 | 0.00E+00 | 3.70E+08 | 0.00E+00 | 8.30E+03 | 3.40E+02 | 1.40E+05 | 3.40E+02 | 1.32E+05 |
|  | 15 | 1.30E+05 | 2.81E+00 | 0.00E+00 | 0.00E+00 | 0.00E+00 | 0.00E+00 | 0.00E+00 | 0.00E+00 |
|  | 16 | 1.70E+05 | 7.20E+08 | 0.00E+00 | 0.00E+00 | 2.60E+02 | 7.90E+04 | 2.60E+02 | 7.90E+04 |
|  | 17 | 1.30E+05 | 6.00E+08 | 0.00E+00 | 0.00E+00 | 2.10E+02 | 9.00E+04 | 2.10E+02 | 9.00E+04 |
|  | 18 | 0.00E+00 | 3.90E+08 | 0.00E+00 | 0.00E+00 | 3.60E+02 | 2.00E+05 | 3.60E+02 | 2.00E+05 |
|  | 19 | 9.00E+04 | 4.00E+08 | 0.00E+00 | 0.00E+00 | 0.00E+00 | 1.20E+05 | 0.00E+00 | 1.20E+05 |
|  | 20 | 2.90E+05 | 5.60E+08 | 0.00E+00 | 0.00E+00 | 3.00E+01 | 5.40E+05 | 3.00E+01 | 5.40E+05 |
|  | Mean | 9.50E+04 | 3.98E+08 | 1.10E+00 | 5.66E+03 | 1.74E+02 | 2.38E+05 | 1.73E+02 | 2.33E+05 |
|  | Log transform of mean |  |  |  |  |  |  | 2.2142563 | 5.05911457 |
| **Artificial feeder** | 1 | 1.30E+05 | 5.40E+08 | 5.00E+01 | 1.90E+04 | 5.00E+03 | 3.30E+06 | 4.95E+03 | 3.28E+06 |
|  | 2 | 6.00E+04 | 3.40E+08 | 1.10E+02 | 1.20E+05 | 5.30E+03 | 2.60E+06 | 5.19E+03 | 2.48E+06 |
|  | 3 | 2.10E+05 | 8.20E+08 | 0 | 0 | 9.00E+03 | 6.40E+06 | 9.00E+03 | 6.40E+06 |
|  | 4 | 2.80E+02 | 2.40E+08 | 0 | 5.10E+04 | 9.00E+03 | 4.30E+06 | 9.00E+03 | 4.25E+06 |
|  | 5 | 2.10E+05 | 1.30E+08 | 1.70E+02 | 9.30E+04 | 7.00E+03 | 2.80E+07 | 6.83E+03 | 2.79E+07 |
|  | 6 | 1.00E+04 | 1.80E+08 | 6 | 9.50E+04 | 0 | 3.00E+06 | -6.00E+00 | 2.91E+06 |
|  | 7 | 2.00E+05 | 5.90E+08 | 4.80E+01 | 5.90E+04 | 4.00E+03 | 2.30E+07 | 3.95E+03 | 2.29E+07 |
|  | 8 | 6.00E+04 | 4.10E+08 | 2 | 6.60E+03 | 1.10E+04 | 8.60E+07 | 1.10E+04 | 8.60E+07 |
|  | 9 | 1.50E+05 | 3.20E+08 | 1.00E+02 | 9.30E+04 | 6.00E+03 | 1.10E+07 | 5.90E+03 | 1.09E+07 |
|  | 10 | 1.40E+05 | 3.90E+08 | 5.10E+01 | 1.00E+04 | 1.00E+03 | 2.70E+07 | 9.49E+02 | 2.70E+07 |
|  | 11 | 1.00E+04 | 1.20E+09 | 1.50E+02 | 3.10E+04 | 1.10E+04 | 1.30E+07 | 1.09E+04 | 1.30E+07 |
|  | 12 | 2.70E+05 | 5.70E+08 | 1.90E+02 | 2.90E+05 | 3.00E+03 | 7.90E+06 | 2.81E+03 | 7.61E+06 |
|  | 13 | 1.10E+05 | 3.00E+08 | 1.00E+02 | 6.50E+04 | 3.30E+03 | 3.50E+04 | 3.20E+03 | -3.00E+04 |
|  | 14 | 2.50E+05 | 1.30E+09 | 3.30E+01 | 3.30E+04 | 3.80E+03 | 9.00E+07 | 3.77E+03 | 9.00E+07 |
|  | 15 | 2.00E+04 | 5.10E+08 | 6.00E+01 | 1.50E+04 | 1.50E+03 | 4.70E+06 | 1.44E+03 | 4.69E+06 |
|  | 16 | 3.10E+05 | 1.00E+09 | 1.20E+02 | 3.60E+04 | 4.70E+03 | 1.40E+07 | 4.58E+03 | 1.40E+07 |
|  | 17 | 4.80E+05 | 4.30E+08 | 9.00E+01 | 3.70E+04 | 2.70E+03 | 3.10E+06 | 2.61E+03 | 3.06E+06 |
|  | 18 | 4.10E+05 | 7.20E+08 | 2.10E+01 | 3.50E+04 | 3.20E+03 | 4.30E+06 | 3.18E+03 | 4.27E+06 |
|  | 19 | 0 | 4.20E+08 | 1.00E+02 | 1.50E+05 | 2.40E+03 | 6.20E+06 | 2.30E+03 | 6.05E+06 |
|  | 20 | 1.60E+05 | 6.20E+08 | 2.10E+01 | 1.90E+03 | 2.60E+03 | 1.80E+07 | 2.58E+03 | 1.80E+07 |
|  | 21 | 9.00E+04 | 3.40E+08 | 1.50E+02 | 3.40E+04 | 1.30E+04 | 1.80E+07 | 1.29E+04 | 1.80E+07 |
|  | 22 | 8.00E+04 | 8.80E+09 | 2.10E+01 | 3.00E+04 | 6.00E+03 | 3.70E+07 | 5.98E+03 | 3.70E+07 |
|  | 23 | 1.80E+05 | 3.20E+08 | 3.00E+02 | 1.80E+05 | 9.00E+03 | 4.40E+07 | 8.70E+03 | 4.38E+07 |
|  | Mean | 1.42E+05 | 8.20E+08 | 7.58E+01 | 5.94E+04 | 4.94E+03 | 1.82E+07 | 4.86E+03 | 1.81E+07 |
|  | Log transform of mean |  |  |  |  |  |  | 3.65639081 | 7.06358567 |
| **Artificial feeder + mixing** | 1 | 9.00E+04 | 5.40E+08 | 6 | 1.00E+04 | 6.00E+03 | 3.10E+07 | 5.99E+03 | 3.10E+07 |
|  | 2 | 0 | 1.80E+04 | 0 | 0 | 0 | 1.80E+04 | 0.00E+00 | 1.80E+04 |
|  | 3 | 9.00E+04 | 2.30E+08 | 2.60E+02 | 3.50E+05 | 4.00E+03 | 4.00E+06 | 3.74E+03 | 3.65E+06 |
|  | 4 | 1.60E+05 | 5.00E+08 | 3.00E+01 | 3.60E+03 | 7.00E+03 | 3.70E+06 | 6.97E+03 | 3.70E+06 |
|  | 5 | 8.00E+04 | 3.50E+08 | 0 | 0 | 4.00E+03 | 7.50E+06 | 4.00E+03 | 7.50E+06 |
|  | Mean | 8.40E+04 | 3.24E+08 | 5.92E+01 | 7.27E+04 | 4.20E+03 | 9.24E+06 | 4.14E+03 | 9.17E+06 |
|  | Log transform of mean |  |  |  |  |  |  | 3.69897028 | 6.35032543 |

| **Supplemental Table 2.** Quantification of virus from CHIKV-infected mosquitoes given different blood feeding treatments, then force salivated and dissected of their bloodmeals. | | | | | | | | | |
| --- | --- | --- | --- | --- | --- | --- | --- | --- | --- |
| **Treatment** | **Mosquito**  **number** | **Bodies** | | **Saliva** | | **Bloodmeals** | | **Difference:**  **bloodmeal-saliva** | |
| **None** |  | Titer | Genome Copy | Titer | Genome Copy | Titer | Genome Copy | Titer | Genome Copy |
|  | 1 | 2.80E+05 | 3.20E+08 | 6.60E+01 | 1.50E+04 | 1.00E+02 | 2.20E+05 | 3.40E+01 | 2.05E+05 |
|  | 2 | 4.10E+04 | 6.90E+07 | 1.00E+01 | 9.10E+03 | 1.00E+01 | 8.10E+05 | 0.00E+00 | 8.01E+05 |
|  | 3 | 1.00E+05 | 8.30E+07 | 3.20E+01 | 2.70E+03 | 2.00E+01 | 2.70E+05 | -1.20E+01 | 2.67E+05 |
|  | 4 | 2.50E+05 | 2.80E+08 | 0 | 0 | 2.90E+02 | 2.10E+05 | 2.90E+02 | 2.10E+05 |
|  | 5 | 3.00E+05 | 2.10E+08 | 1.80E+01 | 1.60E+03 | 8.00E+01 | 5.40E+05 | 6.20E+01 | 5.38E+05 |
|  | 6 | 4.80E+04 | 1.10E+08 | 2 | 0 | 1.10E+02 | 1.80E+05 | 1.08E+02 | 1.80E+05 |
|  | 7 | 3.90E+04 | 7.60E+07 | 8 | 1.10E+03 | 8.00E+01 | 2.30E+04 | 7.20E+01 | 2.19E+04 |
|  | 8 | 4.80E+04 | 1.10E+08 | 2 | 0 | 1.50E+02 | 1.50E+05 | 1.48E+02 | 1.50E+05 |
|  | 9 | 2.70E+05 | 1.40E+08 | 0 | 0 | 3.40E+02 | 1.90E+05 | 3.40E+02 | 1.90E+05 |
|  | 10 | 8.00E+04 | 1.90E+08 | 2 | 0 | 4.20E+02 | 1.30E+05 | 4.18E+02 | 1.30E+05 |
|  | 11 | 2.50E+05 | 1.40E+08 | 6 | 0 | 4.50E+02 | 1.00E+05 | 4.44E+02 | 1.00E+05 |
|  | 12 | 5.20E+04 | 8.90E+07 | 0 | 0 | 4.00E+01 | 3.00E+04 | 4.00E+01 | 3.00E+04 |
|  | 13 | 1.50E+05 | 1.40E+08 | 4 | 0 | 5.90E+02 | 1.20E+06 | 5.86E+02 | 1.20E+06 |
|  | 14 | 1.90E+05 | 1.50E+08 | 0 | 0 | 2.60E+02 | 7.80E+04 | 2.60E+02 | 7.80E+04 |
|  | 15 | 4.60E+04 | 6.50E+07 | 0 | 0 | 1.10E+02 | 6.00E+04 | 1.10E+02 | 6.00E+04 |
|  | 16 | 8.00E+04 | 1.10E+08 | 0 | 0 | 5.00E+01 | 7.90E+04 | 5.00E+01 | 7.90E+04 |
|  | 17 | 5.50E+05 | 4.20E+08 | 1.80E+02 | 2.90E+04 | 2.70E+02 | 2.80E+05 | 9.00E+01 | 2.51E+05 |
|  | 18 | 2.10E+06 | 1.00E+04 | 3.80E+01 | 0 | 1.40E+02 | 0 | 1.02E+02 | 0.00E+00 |
|  | 19 | 4.80E+04 | 1.70E+08 | 0 | 2.00E+04 | 1.00E+01 | 1.70E+05 | 1.00E+01 | 1.50E+05 |
|  | 20 | 0 | 5.30E+07 | 0 | 0 | 0 | 1.40E+05 | 0.00E+00 | 1.40E+05 |
|  | Mean | 2.46E+05 | 1.46E+08 | 1.84E+01 | 3.93E+03 | 1.76E+02 | 2.43E+05 | 1.58E+02 | 2.39E+05 |
|  | Log transform of mean |  |  |  |  |  |  | 2.06679183 | 5.19506467 |
| **Artificial feeder** | 1 | 1.60E+04 | 9.90E+04 | 0 | 0 | 2.00E+02 | 6.50E+05 | 2.00E+02 | 6.50E+05 |
|  | 2 | 1.10E+05 | 4.20E+07 | 0 | 0 | 4.70E+03 | 3.20E+06 | 4.70E+03 | 3.20E+06 |
|  | 3 | 2.50E+05 | 8.00E+07 | 0 | 0 | 1.40E+03 | 2.50E+07 | 1.40E+03 | 2.50E+07 |
|  | 4 | 9.00E+04 | 1.40E+08 | 0 | 1.70E+04 | 1.20E+03 | 3.40E+06 | 1.20E+03 | 3.38E+06 |
|  | 5 | 1.00E+05 | 1.50E+08 | 0 | 2.30E+04 | 7.00E+02 | 4.10E+06 | 7.00E+02 | 4.08E+06 |
|  | 6 | 1.20E+05 | 1.20E+08 | 0 | 0 | 1.30E+03 | 3.10E+06 | 1.30E+03 | 3.10E+06 |
|  | 7 | 3.50E+05 | 2.80E+08 | 2.80E+01 | 2.20E+04 | 4.50E+03 | 8.70E+06 | 4.47E+03 | 8.68E+06 |
|  | 8 | 1.30E+05 | 3.10E+08 | 0 | 0 | 3.80E+03 | 1.80E+07 | 3.80E+03 | 1.80E+07 |
|  | 9 | 4.30E+05 | 2.20E+08 | 2 | 2.10E+03 | 9.00E+03 | 1.00E+07 | 9.00E+03 | 1.00E+07 |
|  | 10 | 2.30E+05 | 4.40E+08 | 1.00E+01 | 2.00E+04 | 7.50E+04 | 1.70E+07 | 7.50E+04 | 1.70E+07 |
|  | 11 | 1.30E+05 | 1.00E+08 | 2.20E+01 | 5.60E+02 | 1.10E+03 | 8.30E+06 | 1.08E+03 | 8.30E+06 |
|  | 12 | 3.30E+05 | 9.00E+07 | 0 | 0 | 2.50E+03 | 1.90E+06 | 2.50E+03 | 1.90E+06 |
|  | 13 | 1.00E+06 | 4.40E+08 | 0 | 0 | 7.00E+04 | 2.20E+06 | 7.00E+04 | 2.20E+06 |
|  | 14 | 3.90E+05 | 7.70E+08 | 3.00E+02 | 3.30E+05 | 2.40E+03 | 1.80E+07 | 2.10E+03 | 1.77E+07 |
|  | 15 | 1.70E+05 | 1.90E+08 | 4 | 0 | 3.30E+03 | 6.40E+06 | 3.30E+03 | 6.40E+06 |
|  | 16 | 1.30E+05 | 2.30E+08 | 6 | 1.00E+03 | 3.00E+02 | 1.10E+07 | 2.94E+02 | 1.10E+07 |
|  | 17 | 3.40E+05 | 1.60E+08 | 4 | 1.80E+04 | 5.10E+03 | 2.00E+06 | 5.10E+03 | 1.98E+06 |
|  | 18 | 2.70E+06 | 3.10E+08 | 5.00E+01 | 2.90E+04 | 7.20E+04 | 8.30E+06 | 7.20E+04 | 8.27E+06 |
|  | 19 | 2.00E+05 | 1.10E+08 | 0 | 0 | 1.40E+03 | 6.20E+07 | 1.40E+03 | 6.20E+07 |
|  | 20 | 1.40E+05 | 1.50E+08 | 1.20E+01 | 2.40E+03 | 2.30E+03 | 1.20E+07 | 2.29E+03 | 1.20E+07 |
|  | 21 | 1.60E+05 | 1.50E+08 | 1.20E+01 | 1.60E+04 | 3.50E+03 | 3.00E+07 | 3.49E+03 | 3.00E+07 |
|  | 22 | 1.40E+05 | 2.20E+08 | 0 | 0 | 1.10E+03 | 1.00E+07 | 1.10E+03 | 1.00E+07 |
|  | 23 | 4.00E+04 | 1.90E+08 | 2 | 1.80E+03 | 8.00E+02 | 7.80E+06 | 7.98E+02 | 7.80E+06 |
|  | 24 | 9.00E+04 | 1.20E+08 | 0 | 6.40E+02 | 5.00E+02 | 4.20E+06 | 5.00E+02 | 4.20E+06 |
|  | 25 | 0 | 1.30E+08 | 0 | 0 | 0 | 1.30E+06 | 0.00E+00 | 1.30E+06 |
|  | Mean | 3.11E+05 | 2.06E+08 | 1.81E+01 | 1.93E+04 | 1.07E+04 | 1.11E+07 | 1.07E+04 | 1.11E+07 |
|  | Log transform of mean |  |  |  |  |  |  | 3.41899626 | 6.82390797 |
| **Artificial feeder + mixing** | 1 | 1.90E+05 | 2.00E+08 | 1.40E+01 | 4.40E+03 | 4.00E+03 | 3.40E+06 | 3.99E+03 | 3.40E+06 |
|  | 2 | 1.10E+05 | 1.20E+08 | 1.40E+01 | 3.70E+04 | 5.00E+03 | 6.00E+06 | 4.99E+03 | 5.96E+06 |
|  | 3 | 7.50E+05 | 5.60E+08 | 4.60E+01 | 6.60E+04 | 11000 | 4.00E+06 | 1.10E+04 | 3.93E+06 |
|  | 4 | 1.10E+05 | 1.00E+08 | 8 | 6.20E+03 | 2.00E+03 | 8.90E+06 | 1.99E+03 | 8.89E+06 |
|  | 5 | 1.10E+05 | 9.40E+07 | 1.60E+01 | 5.50E+03 | 3.00E+03 | 2.20E+06 | 2.98E+03 | 2.19E+06 |
|  | 6 | 9.00E+04 | 1.70E+08 | 6 | 5.80E+03 | 1.00E+03 | 2.40E+06 | 9.94E+02 | 2.39E+06 |
|  | 7 | 1.00E+05 | 2.90E+08 | 1.10E+02 | 5.70E+04 | 0 | 5.90E+06 | -1.10E+02 | 5.84E+06 |
|  | 8 | 1.40E+05 | 2.50E+08 | 2.80E+01 | 2.00E+04 | 0 | 1.70E+06 | -2.80E+01 | 1.68E+06 |
|  | 9 | 9.00E+04 | 2.40E+08 | 4.40E+01 | 3.90E+04 | 3.00E+03 | 3.50E+06 | 2.96E+03 | 3.46E+06 |
|  | 10 | 1.50E+05 | 1.90E+08 | 0 | 0 | 0 | 1.60E+06 | 0.00E+00 | 1.60E+06 |
|  | 11 | 1.00E+05 | 1.40E+08 | 1.00E+01 | 2.20E+03 | 1.00E+03 | 8.80E+06 | 9.90E+02 | 8.80E+06 |
|  | 12 | 2.70E+05 | 1.80E+08 | 4.20E+01 | 6.90E+03 | 1.80E+04 | 1.60E+07 | 1.80E+04 | 1.60E+07 |
|  | 13 | 1.30E+06 | 1.30E+09 | 8 | 7.70E+03 | 4.60E+04 | 9.90E+06 | 4.60E+04 | 9.89E+06 |
|  | 14 | 2.80E+05 | 3.30E+08 | 1.60E+01 | 3.40E+03 | 0 | 3.70E+06 | -1.60E+01 | 3.70E+06 |
|  | 15 | 2.00E+04 | 3.40E+06 | 4 | 4.50E+03 | 0 | 1.70E+04 | -4.00E+00 | 1.25E+04 |
|  | 16 | 1.70E+05 | 1.80E+08 | 1.40E+01 | 1.60E+04 | 0 | 4.20E+06 | -1.40E+01 | 4.18E+06 |
|  | 17 | 1.50E+05 | 2.20E+08 | 3.80E+01 | 1.30E+04 | 1.00E+03 | 3.60E+06 | 9.62E+02 | 3.59E+06 |
|  | 18 | 3.10E+05 | 1.40E+08 | 1.20E+01 | 2.60E+03 | 2.00E+04 | 3.40E+07 | 2.00E+04 | 3.40E+07 |
|  | 19 | 9.00E+04 | 1.80E+08 | 1.20E+01 | 3.80E+04 | 0 | 1.00E+06 | -1.20E+01 | 9.62E+05 |
|  | 20 | 9.00E+04 | 7.50E+08 | 4.00E+01 | 9.60E+04 | 0 | 1.10E+06 | -4.00E+01 | 1.00E+06 |
|  | 21 | 1.60E+05 | 2.80E+08 | 5.40E+01 | 3.60E+04 | 3.00E+03 | 7.20E+06 | 2.95E+03 | 7.16E+06 |
|  | 22 | 7.20E+05 | 5.70E+08 | 2.00E+02 | 7.50E+04 | 4.50E+04 | 8.40E+07 | 4.48E+04 | 8.39E+07 |
|  | 23 | 6.00E+04 | 1.00E+08 | 2.60E+01 | 1.30E+04 | 0 | 2.00E+07 | -2.60E+01 | 2.00E+07 |
|  | 24 | 2.50E+05 | 3.60E+08 | 2.60E+01 | 1.50E+04 | 1.70E+04 | 1.80E+07 | 1.70E+04 | 1.80E+07 |
|  | 25 | 6.00E+04 | 1.30E+08 | 8 | 1.00E+03 | 5.00E+03 | 2.10E+07 | 4.99E+03 | 2.10E+07 |
|  | Mean | 2.35E+05 | 2.83E+08 | 3.18E+01 | 2.28E+04 | 7.40E+03 | 1.09E+07 | 7.37E+03 | 1.09E+07 |
|  | Log transform of mean |  |  |  |  |  |  | 3.73907067 | 6.65855854 |
